# Effect of AuPd Bimetal Sensitization on Gas Sensing Performance of Nanocrystalline SnO_2_ Obtained by Single Step Flame Spray Pyrolysis

**DOI:** 10.3390/nano9050728

**Published:** 2019-05-10

**Authors:** Valeriy Krivetskiy, Konstantin Zamanskiy, Artemiy Beltyukov, Andrey Asachenko, Maxim Topchiy, Mikhail Nechaev, Alexey Garshev, Alina Krotova, Darya Filatova, Konstantin Maslakov, Marina Rumyantseva, Alexander Gaskov

**Affiliations:** 1 Department of Chemistry, Lomonosov Moscow State University, Leninskie gory 1/3, 119234 Moscow, Russia; asandrey@yandex.ru (A.A.); maxtopchiy@ya.ru (M.T.); m.s.nechaev@org.chem.msu.ru (M.N.); garshev@inorg.chem.msu.ru (A.G.); alinakrotova1996@mail.ru (A.K.); gak1.analyt@gmail.com (D.F.); nonvitas@gmail.com (K.M.); roum@inorg.chem.msu.ru (M.R.); gaskov@inorg.chem.msu.ru (A.G.); 2 Faculty of Materials Sciences, Lomonosov Moscow State University, Leninskie gory 1/3, 119234 Moscow, Russia; zambahrs97@gmail.com; 3 Udmurt Federal Research Center of UB RAS, Laboratory of Atomic Structure and Surface Analysis, Kirova 132, 426000 Izhevsk, Russia; beltukov.a.n@gmail.com; 4 A.V. Topchiev Institute of Petrochemical Synthesis, Russian Academy of Sciences, Leninsky Prospect 29, 119991 Moscow, Russia

**Keywords:** nanocrystalline SnO_2_, semiconductor gas sensor, bimetal sensitization, flame spray pyrolysis

## Abstract

Improvement of sensitivity, lower detection limits, stability and reproducibility of semiconductor metal oxide gas sensor characteristics are required for their application in the fields of ecological monitoring, industrial safety, public security, express medical diagnostics, etc. Facile and scalable single step flame spray pyrolysis (FSP) synthesis of bimetal AuPd sensitized nanocrystalline SnO_2_ is reported. The materials chemical composition, structure and morphology has been studied by XRD, XPS, HAADFSTEM, BET, ICP-MS techniques. Thermo-programmed reduction with hydrogen (TPR-H_2_) has been used for materials chemical reactivity characterization. Superior gas sensor response of bimetallic modified SnO_2_ towards wide concentration range of reducing (CO, CH_4_, C_3_H_8_, H_2_S, NH_3_) and oxidizing (NO_2_) gases compared to pure and monometallic modified SnO_2_ is reported for dry and humid gas detection conditions. The combination of facilitated oxygen molecule spillover on gold particles and electronic effect of Fermi level control by reoxidizing Pd-PdO clusters on SnO_2_ surface is proposed to give rise to the observed enhanced gas sensor performance.

## 1. Introduction

High sensitivity and low detection limits of semiconductor metal oxide gas sensors define attempts of their application in the field of medical diagnostics [1] and monitoring [2], environmental [3] and building air quality control [4], as well as detection of highly poisonous, toxic and explosive compounds at extreme low concentrations [5]. The expansion of metal oxide sensors into the field various consumer devices, internet of things and artificial olfaction systems is associated with the novel signal processing methods [6,7] for gas sensor arrays [8] or even single sensors, operating in modulated working temperature mode [9]. Such an approach not only allows for improved selectivity of response, detection limits and working concentration range, but also mitigates sensor drift effects [10,11]. It has been demonstrated, that using of chemically modified metal oxides, particularly nanocrystalline SnO_2_, with catalytic components, which are known to improve sensor response, provides a beneficial effect on the accuracy of the detection (both identification and quantification) [12]. Thus, reliable detection of analytes below ppm concentration level in real atmospheric conditions (variable humidity and temperature) require further improvements in sensors’ sensitivity and lower detection limits. The use of bimetallic nanoparticles in order to sensitize semiconductor metal oxide gas sensors has attracted more and more attention lately [13]. Particularly gold-containing nanoparticles with Pt-group metals have been reported to provide profound improvements in certain gases detection [14,15,16,17,18]. In the limited number of papers the observed effect has been suggested to arise from combination of catalytic and electronic sensibilization effects on the metal oxide surfaces. Further improvements of synthetic procedures of such nanocomposites are of high demand apart from extensive research on this sensibilization effect origins and its manifestation on the extended number of gases. Currently bimetallic nanoparticle (NP) functionalized semiconductor metal oxides are obtained in a two step process: either separately prepared bimetallic nanoparticles or noble metal precursors with further reduction are deposited on the previously synthesized metal oxide matrix. Such a procedure is time and labor consuming and bears risks of introduction of impurities in the final nanocomposite or deviations of NP content. Insufficient sensor parameters repeatability from sample to sample, arising from such causes, requires sophisticated calibration models and increases device cost [19].

An alternative way of metal oxide gas sensor production using metal organic precursors in flame spray pyrolysis (FSP) process has been recently developed [20]. It allows for high precision of components content control and homogeneity of their distribution in the final material structure. The use of shadow masking allows mass production of sensors in a single synthetic step with potentially high reproducibility of gas sensor parameters [21]. In this work we report superior gas sensing properties of bimetal—Au and Pd—modified nanocrystalline SnO_2_, obtained in single step via FSP technique, towards a wide spectrum of gases with different chemical nature.

## 2. Materials and Methods

### 2.1. Materials Synthesis

All materials were synthesized via flame spray pyrolysis technique. The custom made apparatus design (Figures S1 and S2) and synthesis protocol were based on the earlier reports of pioneering researchers [22]. The following metal organic precursors were used for synthesis: tin (II) ethylhexanoate (Sigma-Aldrich, 98%, St. Louis, MO, USA), palladium acetyacetonate (Sigma-Aldrich, 98%) and (1,3-bis(2,6-diisopropylphenyl)-1,3-diazepan-2-ylidene) gold(I) acetate. Toluene (99.5%) was utilized as fuel. Metal organic precursors were mixed with fuel in a 1:4 ratio by volume. A syringe pump (KD scientific, Holliston, MA, USA) was used to supply the mixture to the spray nozzle at a rate of 3 mL/min. The mixture was sprayed by 3 L/min oxygen (99.95%) flow at 3 bar pressure drop. The materials were collected by GF/A glass fiber filters (GE Whatman, Sigma-Aldrich, St. Louis, MO, USA), located 90 cm above the nozzle with the aid of an ISP 250 C vacuum pump (Anest Iwata, Yokohama, Japan). Powder samples were collected manually from the filter surface and then used as prepared for analysis and sensor manufacturing.

### 2.2. Gas Sensor Fabrication

Gas sensors were fabricated by deposition of gas sensitive materials on top of the polycrystalline Al_2_O_3_ substrates, which had Pt contacts for resistance measurement on one side and a Pt heating element on the other. The substrate dimensions were 2 mm × 2 mm × 0.15 mm. Materials were deposited in a form of a paste, which was created with the use of α-terpineol as a binder. After the deposition the substrate was heated up to 500 °C and kept at this temperature for 12 h in the laboratory ambient air atmosphere for the binder to completely evaporate and possible organic contaminants to burn out. As prepared sensors were used for further measurements.

### 2.3. Chemical Composition, Structure, Morphology and Reactivity

Synthesized materials phase composition was studied by X-Ray diffraction (XRD) with the use of Rigaku D/MAX 2500 diffractometer (Rigaku, Tokyo, Japan) with Cu Ka radiation (λ = 1.5406 Å) in 20–80 of 2φ range. Joint Committee on Powder Diffraction Standards (JCPDS) database was used for phase analysis. Grain size was calculated by the Sсherrer equation with the use of α-Al_2_O_3_ as a standard.

Chemical composition of the synthesized samples was studied by inductively coupled plasma mass-spectrometry (ICP-MS) technique. Measurements were performed on an Agilent 7500 C inductively coupled plasma quadrupole mass spectrometer (Agilent Technologies, Tokyo, Japan). The spectrometer was controlled with a PC using the ChemStation (version G1834B) software package (Agilent Technologies). Measurements were performed for isotopes 197 Au, 105 Pd and 106 Pd. For the determination of Pd and Au, 1 mL of aqua regia were added to the weighed 0.0020 g of the samples and left for 10 h at room temperature. Then solutions were heated to remove nitrous oxides and adjusted to volume with 1% HCl (Merck, Darmstadt, Germany) after cooling.

For the clarification of tin to palladium or gold ratio about 1 mg of some samples were digested in a mixture of 0.8 mL aqua regia and 0.7 mL of HF (Merck, Darmstadt, Germany) in an autoclave in a microwave oven MARS 5 microwave accelerated reaction system with 12 XP 1500 Plus high-pressure vessels (CEM, Matthews, NC, USA). Samples were irradiated for 70 min (working frequency of the system was 2455 MHz, radiated power was 500 W and the temperature was 220 °C).

For the ICP-MS analysis all the resulting solutions were diluted with 1% HCl. Standard solutions of Au and Pd were prepared by serial dilution from the multielement standard solution (High-Purity Standards, Charleston, SC, USA) with concentrations of the determined elements of 10 mg L^−1^. The solution of the control sample was used for the measurement of the background signal. The results of the analysis were obtained with the relative standard deviation (RSD) being less than 5%.

Axis Ultra DLD (Kratos Analytical, Manchester, UK) spectrometer was used to study chemical state of the elements via X-ray photoelectron spectroscopy (XPS) technique. High resolution XPS spectra were acquired with monochromatic Al Kα radiation at 40 eV pass energy using charge compensation. Spectra were charge referenced using Sn3d_5/2_(SnO_2_) line—487.2 eV.

High angle annular dark field scanning transmission electron microscopy (HAADFSTEM) images and energy-dispersive X-ray spectroscopy (EDX) maps were acquired using Libra 200 MC (Carl Zeiss, Jena, Germany) microscope operated at 200 kV. HAADF images were obtained using Fischione detector (E.A. Fischione Instruments, Inc., Pittsburgh, PA, USA) with a condenser aperture of 20 mm. EDX maps were collected using X/MAX 80 TEM (Aztec, Oxford Instruments, Abingdon, UK) detector.

MicroMeritics ChemiSorb 2750 device (Micromeritics, Norcross, GA, USA) was used for Brunauer-Emmett-Teller (BET) surface measurements according to single point low temperature N_2_ adsorption protocol. The same apparatus was involved in temperature programmed study of materials reduction with hydrogen (TPR-H_2_). According to this procedure the materials were conditioned at 500 °C in the flow of dry synthetic air for 30 min, cooled down to room temperature and then subjected to the 10 °C/min temperature ramp in the flow of H_2_/Ar mixture (8% by volume) until 900 °C temperature was reached.

### 2.4. Gas Sensor Properties

Gas sensor properties of the synthesized materials were measured at 1 Hz frequency of sensor layer resistance values acquisition in DC mode with the use of flow-through sealed sensor chamber, similar to what was reported previously [23]. The limit of detection for the manufacture sensors was estimated by linear extrapolation of concentration dependence of gas sensor response to the values, equal to noise signal. Sensor noise was estimated as response, calculated based on sensor resistance fluctuations at lowest measured concentration of target gas, multiplied by three.

## 3. Results and Discussion

### 3.1. Nanocomposites Morphology and Structure

Only a single phase of rutile structure SnO_2_ (JCPDS Card No 41-1445) has been detected by XRD in all synthesized materials (Figure S3), since the noble metal content in the synthesized nanocomposites is below of the method detection limit. Morphological and structural properties of synthesized materials, based on XRD and BET results, are summarized in Table 1.

A good correlation between loaded and actual Au and Pd elemental content in the synthesized nanocomposites was found by ICP MS technique. It can be seen, that addition of noble metal components leads to notable increase of effective surface area, which is accompanied by slight decrease of grain size. This effect is related to hampered intergrain mass transfer due to surface impurities and is well known from previous studies [24]. Particle size distribution, calculated on the basis of TEM micrographs, support the Scherrer formula calculations (Figures S4–S7). Somewhat wider distribution of particle sizes can be traced in case of noble metal modified samples, compared to pure SnO_2_.

**Figure 1:**
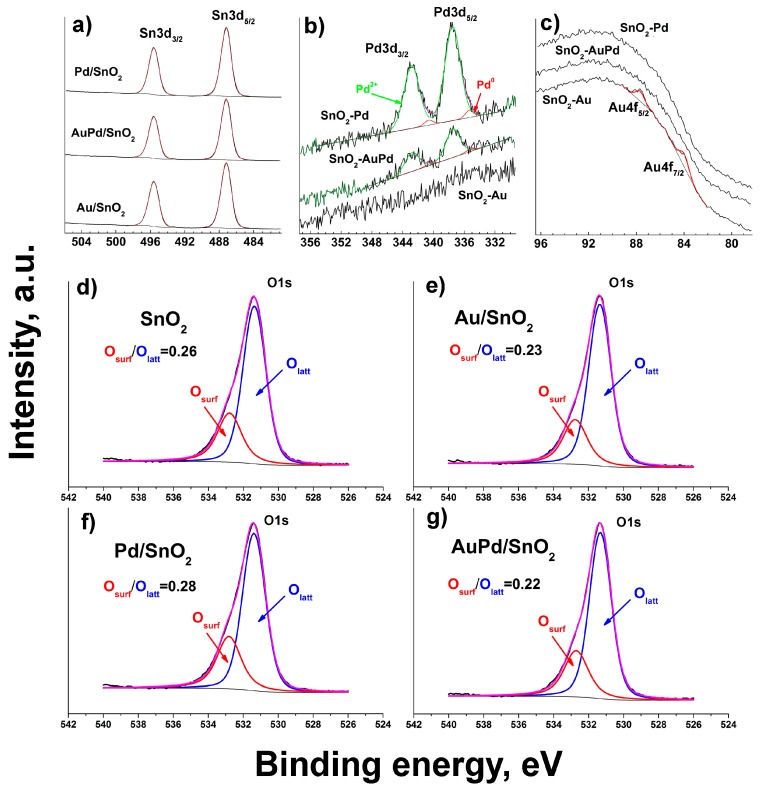
(**a**) Sn3d high resolution XP spectra of metal-loaded materials, (**b**) Pd3d lines and (**c**) Au4f lines for the same samples, (**d**–**g**) O1s peaks for all synthesized materials.

XP spectra reveal Sn3d line Sn3d5/2 (487.2 eV) corresponding to SnO_2_ in all synthesized materials (Figure 1a) [25].

The energy of Pd3d_5/2_ peak (337.4–337.5) (Figure 1b), observed for Pd/SnO_2_ and AuPd/SnO_2_ samples, corresponds to Pd^2+^ cations, but however is higher than the one known for PdO (336.7 eV) [26]. Deconvolution of Pd3d XP spectra allows the assumption of a small fraction of metallic Pd in the samples, around 4% of total Pd content. However, due to low intensity of Pd3d peaks it is hard to make a firm statement on that matter. Gold 4f lines are observed only in the Au/SnO_2_ sample (Figure 1c), while in the AuPd/SnO_2_ they are not present, probably due to the low Au content. The sensitivity of the XPS technique towards Au is additionally lowered in the given samples due to overlapping of Au4f lines by more intense and broad Sn4p line. The observed energy of Au4f_7/2_ peak (84.0 eV) corresponds to metallic gold [27]. O1s XP lines for synthesized materials (Figure 1d–g) reveal special features, originating due to metal modification of SnO_2_ surface. The observed lines can be described by two components, one of which (531.4 eV) is common for all obtained materials and corresponds to SnO_2_ lattice O^2−^ ions. Only in the case of bimetallic modified sample AuPd/SnO_2_ is it slightly shifted to 531.3 eV, indicating a kind of stronger electronic interaction between the noble metal and metal oxide components, than in case of monometallic modified samples. The other component (532.8 eV) is usually attributed to various surface oxygen species, originating from ambient oxygen chemisorption and dissociation [28]. This component again is shifted (532.7 eV) in case of bimetallic loaded sample, which corresponds to modified chemical environment for surface-related oxygen species. Moreover, the O1s XPS spectra component peak areas indicate that the atomic ratio of surface to lattice oxygen significantly increases upon Pd-metal modification of SnO_2_. This can be attributed to reported previously facilitated formation of surface hydroxyl groups upon functionalization of SnO_2_ with Pd [14]. Most probably it occurs due to partial oxidation of metallic Pd on the SnO_2_ grain surface. In the case of Au/SnO_2_ and AuPd/SnO_2_ this ratio is lower than in case of pure SnO_2_. This may occur due to the reported enhancement of oxygen dissociative adsorption due to catalytic spillover effect of gold particles, distributed over tin dioxide grains [29]. Such new sites of O_2_ molecule adsorption may affect the distribution of surface negative charge, concentrating ionized oxygen species in the local area in proximity around gold particles. Thus, more distant from gold particles oxygen chemisorption sites become inactive. Additionally, the active surface oxygen forms in the case of Au-modified SnO_2_ will be discussed below during TPR-H_2_ spectra analysis.

EDX mapping revealed very uniform distribution of Pd and Au over the structure of the obtained bimetal modified nanocomposite. In combination with low noble metal content it did not allow us to find any separate bimetallic or monometallic particles during the TEM study of the materials. However, the reconstruction of EDX spectra and elemental contents for local areas on the total maps (Table 2) revealed some metal-specific differences in distribution.

**Figure 2:**
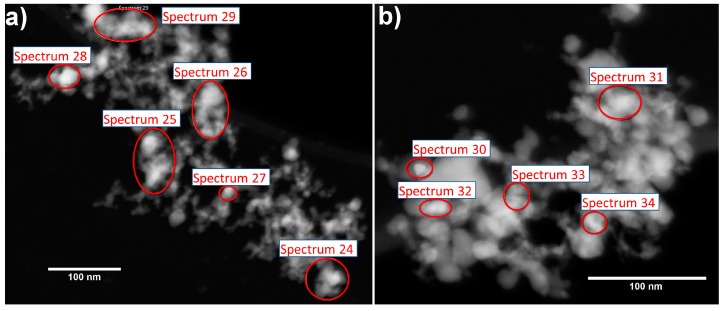
High angle annular dark field scanning transmission electron microscopy (HAADF-STEM) images of different parts of AuPd/SnO_2_ sample, (**a**) and (**b**), used during EDX mapping procedure. The obtained EDX maps are not informative due to low Au and Pd content and homogeneous distribution of elements. Local spots, for which spectra were reconstructed from total map spectrum, are designated. Analysis of Au and Pd content at local spots of EDX maps (Table 2) allows conclusions to be made on Pd and Au distribution over the nanocomposite structure.

Due to low Pd content the detected concentration of this element in all local spots is around 3 at.% or less. It indicates very uniform and homogeneous distribution of Pd over the nanocomposite structure in accordance with the previous report on flame-made Pd/SnO_2_ material [30]. The XPS data of the present study in combination with elemental mapping support the idea, that a significant part of the Pd occupy cationic positions in SnO_2_ lattice in the grains surface in the form of Pd^2+^ cations. Only a small fraction of Pd, introduced into the AuPd/SnO_2_ nanocomposite, form PdO-like clusters, which can be partially reduced to metallic state. Contrarily, in case of Au, very high concentrations can be detected at local spots (see spectrum 30 on Figure 2 and Table 2) of AuPd/SnO_2_ nanocomposite. It indicates formation of 5–10 nm size gold nanoparticles alongside with sub-nm size metal clusters, distributed homogeneously over the SnO_2_ surface. The applied techniques did not allow the detection any bimetallic particles formed during AuPd/SnO_2_ material synthesis due to two main reasons—very low noble metal content and very homogeneous distribution of metallic form elements in, probably, sub-nm size clusters.

### 3.2. Chemical Reactivity

**Figure 3:**
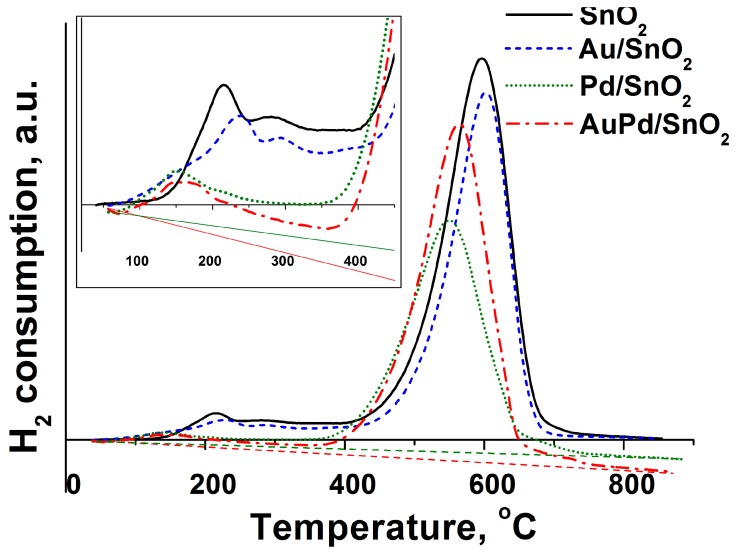
Spectra of H_2_ consumption during thermo-programmed reduction by hydrogen of synthesized materials. Spectra are not normalized to the mass or total surface area of samples due to the excessive hydrogen absorption by metallic Pd particles.

Spectra of H_2_ consumption during thermo-programmed reduction of the materials are given in Figure 3.

The pure SnO_2_ spectrum resembles the one for sol-gel-produced tin dioxide and reflects the process of hydrogen consumption separately by active surface chemisorbed oxygen species at low temperatures below 450 °C and reduction of SnO_2_ to metallic tin with the maximum at 600 °C [31]. Low temperature region of H_2_ consumption spectrum by pure SnO2 also contains local maxima at 215 and 280 °C, which correspond to different forms of mobile surface oxygen. Introduction of Pd in the nanocomposites chemical composition shifts both low and high temperature H_2_ consumption maxima to lower temperature values. This effect is usually attributed to a catalytic effect of metallic Pd particles on the surface of SnO_2_ grains, which originates in H_2_ dissociative adsorption and interaction with surface and lattice oxygen species through spillover effect [32]. The temperature of the onset of H_2_ consumption in the case of all metal-modified samples is also lower (50–60 °C), compared to pure SnO_2_ (140–150 °C). In case of the Pd-containing samples it may reflect the reduction of PdO clusters to metallic Pd, while for Au-modified materials—the presence of additional highly active form of chemisorbed oxygen on the surface of SnO_2_ grains. Interestingly, the baseline of the spectra of Pd-containing materials have negative inclines which can only be associated with additional hydrogen release with the growth of the sample temperature. This hydrogen may be accumulated by Pd particles during the sample preparation stage due to high hydrogen solubility in the metallic palladium. Although XPS have shown only minute amounts of Pd in metallic state, the PdO clusters may be easily reduced to Pd^0^ state when the gas flow in the quartz tube with nanocomposite is switched from dry synthetic air to the mixture of H_2_ in Ar prior to the application of the temperature ramp. Usually the experiment is started only when TCD readings are stable in time, so we can assume that by this moment the equilibrium is reached in PdO reduction at room temperature by H_2_/Ar mixture. Such baseline incline is not observed in case of material, modified solely with gold, which is not absorbing hydrogen. Moreover, a notable shift of H_2_ consumption low temperature maxima to higher temperatures (215 to 240 °C and 280 to 295 °C) is observed for Au/SnO_2_ (see inset on Figure 3), which indicates an increase of activation energy of materials surface reduction reaction. This increase may be associated with hampered surface mobility and localization of chemisorbed oxygen species on the SnO_2_ grains surface in the ternary phase interface “Au-SnO_2_-gas”. Formation of such, already mentioned above, localized species is likely due to their electrophilic nature. Formation of such species may be activated through interaction between gold particles and adjacent oxygen vacancies on the surface of SnO_2_ grains [33].

Due to the hydrogen absorption in the Pd clusters only the position of H_2_ consumption maxima are relevant, while calculations and comparison of total H_2_ consumed quantity can not give useful information in this study.

### 3.3. Gas Sensor Properties

**Figure 4:**
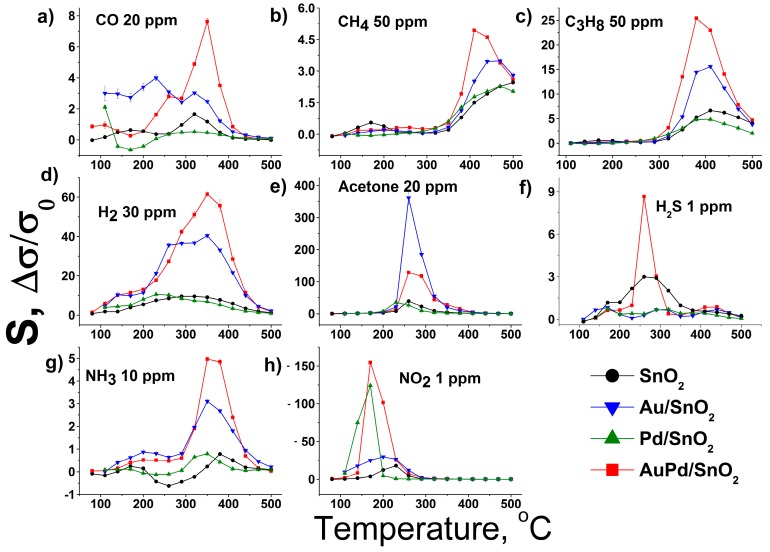
Dependence of gas sensor response of synthesized materials on sensor working temperature towards (**a**) CO 20 ppm, (**b**) CH_4_ 50 ppm, (**c**) C_3_H_8_ 50 ppm, (**d**) H_2_ 30 ppm, (**e**) acetone ppm, (**f**) H_2_S 1 ppm, (**g**) NH_3_ 10 ppm, (**h**) NH_3_ 10 ppm in dry air.

**Figure 5:**
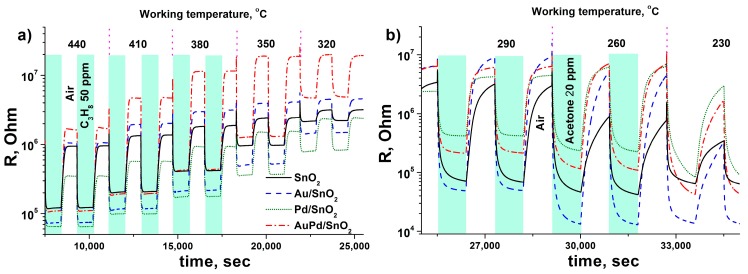
Resistance transients during the measurements of sensor response of synthesized materials at different working temperatures towards (**a**) C_3_H_8_ 50 ppm and (**b**) acetone 20 ppm.

Profiles of temperature dependence of the sensor response of synthesized materials towards a set of gases is given in Figure 4a–h. As can be seen, the maximum sensor response is observed at different working temperature for each gas used in the test. This effect is related to different reactivity of gases, which depends on their molecular structure and surface oxidation mechanism [31,34]. Particularly, the CO molecule can be oxidized via Langmuir–Hinshelwood mechanism by SnO_2_ surface chemisorbed oxygen species [31]. This process is related to the formation of high temperature sensor response at 320–350 °C and is prominent in case of Au-modified samples due to facilitated O_2_ chemisorption [34]. On the contrary Pd-modified SnO_2_ demonstrates low temperature sensor response maximum towards this gas, which is also reported in literature [35,36] and considered to be attributed to Eley–Rideal oxidation mechanism on the partially oxidized Pd clusters on the SnO_2_ grain surface [31]. The local sensor response maximum at around 200 °C in case of the pure SnO_2_ material should be attributed to the sensitivity to the residual water content in the certified CO gas bottle [37], as the reference air has water content below 10 ppm. This notion is supported by local minimum of sensor response for metal-modified samples at the same working temperatures, In the case of Pd/SnO_2_ sample this process is even associated with negative sensor response formation at 140–200 °C working temperature range. Such behavior of SnO_2_-based materials during CO detection in humid conditions has been reported previously and was associated with formation of electron-accepting carbonate groups $CO_{3}^{2-}$ on the SnO_2_ surface [38]. Propane and especially methane, being more chemically inert molecules compared to CO, demonstrate maximum sensor response at considerable higher sensor working temperatures in accordance with previously reported studies [39,40,41]. Hydrogen is characterized by single sensor response maximum as well, which is, however is much broader, compared to other gases, due to the high chemical reactivity and diffusion rate of this gas [42,43]. The maximum sensor response of acetone, on the contrary, is very narrow and shifted to lower temperatures, representing a complex interplay of numerous intermediate acetone oxidation products complete burning (at higher sensor working temperatures) and their strong chemisorption (at lower operating temperatures) [44]. Hydrogen sulfide detection pattern for pure SnO_2_ material resembles results, obtained in the previous studies—broad gas sensor response maximum at 200–300 °C operating temperatures [45,46]. Contrary to the previous reports [34,47,48,49,50] the monometallic-modified SnO_2_ demonstrated a drop of response towards H_2_S, compared to pure metal oxide in the all tested working temperature ranges. The observed deterioration of gas sensor performance should be connected to the metal surface poisoning with intermediates of H_2_S conversion—atomic sulfur and sulfate ions or even palladium sulfide [51,52]. Interestingly, the bimetallic-modified material shows prominent improvement in response towards H_2_S, which can be connected to the prevented poisoning of metallic surface due to partial alloying of the metal components and manifestation of catalytic performance of the bimetallic system [53]. This matter requires a separate study with the use of in situ spectroscopy techniques. The sensor response temperature dependence pattern in case of ammonia detection is characterized by local minimum at 250 °C. It is observed for all tested materials and associated with negative response values in the case of pure SnO_2_. Such gas sensor behavior is attributed to in situ NO formation, which adsorbs on the surface of SnO_2_ as electrophilic nitrite and nitrate species [23,54]. The elevation of sensor working temperature leads to NOx species desorption due to competition for adsorption sites with abundant ambient oxygen molecules [31]. That is why the maximum of sensor response towards NO_2_ for all tested materials is observed in 170–250 °C temperature range. In most cases gold and bimetal modified SnO_2_ exhibit the same pattern of sensitivity, as the original unmodified tin dioxide. Particularly, in the case of reducing gases—CO, CH_4_, C_3_H_8_, H_2_, NH_3_, H_2_S—maximum of sensor response is recorded at nearly the same working temperatures for pure SnO_2_, Au/SnO_2_ and AuPd/SnO_2_ samples. However, the bimetal modified sample demonstrates significantly higher response values towards given gases, compared to other nanocomposites. The gas sensor resistance transients, recorded during response measurements towards propane (Figure 5a) indicate that an excellent response in the case of bimetal loaded sample in achieved due to increase of the material’s baseline resistance in the flow of dry clean air, rather than profound drop of resistance in the presence of reducing gas. The latter is the case for acetone sensing (Figure 5b), during which a nanocomposite, modified with single metal—gold—demonstrates several times greater sensor response compared to other samples, including SnO_2_ with bimetallic modification. Enhanced gas sensor performance of gold-modified SnO_2_ has been assigned to activated oxygen chemisorption and dissociation via spillover effect [29,55]. The latter circumstance may dominate the formation of the sensor response in the case of interaction with highly active reducing gas molecules. The excel of Au/SnO_2_ over AuPd/SnO_2_ in acetone detection can be explained due to higher Au content and, hence, elevated concentration of active oxidation sites on the surface. The profound response improvement towards acetone in case of Au-modified SnO_2_ and other metal oxides has been reported previously [14,15,34,56,57]. Although the direct comparison of laboratory-made gas sensor sample performance is complicated due to different chip design, humidity conditions, material chemical composition and other measurement parameters, we can conclude that presented here acetone sensitivity of Au/SnO_2_ material towards acetone matches one of the best reported performances of material with close Au content and far more complicated synthesis protocol [58]. The higher sensor response of bimetal modified AuPd/SnO2 material towards other, less active reducing gases becomes clear from the analysis of the sensitivity towards NO_2_.

Maximum sensor response towards oxidizing gas—NO_2_—is also observed in the case of bimetal modified SnO_2_. However, Pd modified tin dioxide demonstrates very similar behavior with profound response towards NO_2_ at low operating temperatures. High sensitivity of semiconductor metal oxides towards NO_2_ upon Pd modification has been reported for systems with various chemical composition and morphology [59,60,61,62]. In case of Pd-clusters formation on SnO_2_ surface their ability to easily switch from reduced to oxidized state plays major role in gas sensor performance. In the case of oxidizing gas NO_2_ complete oxidation of Pd clusters to PdO state generates electron depleted regions in the SnO_2_ near surface layer around in accordance with Fermi level control mechanism, leading to the profound rise of electrical resistance [63,64]. In this sense conditions of dry air at elevated temperatures may be considered as reducing, compared to the presence of 1 ppm of NO_2_. Hence, some fraction of PdO clusters in such conditions return at partially oxidized or even reduced states. It may be speculated, that in the case of bimetallic modified SnO_2_ activation of surface oxygen species through spillover effect by metallic Au leads to more complete oxidation of PdO clusters, resulting in the observed higher baseline resistance of the material. The close proximity of Pd and Au components on the SnO_2_ surface facilitate this sensibilization mechanism. It should be noted, that Au/SnO_2_ material also demonstrates improved sensitivity towards NO_2_, compared to pure SnO_2_. Such behavior has also been observed in previous studies with wet-procedure obtained Au/SnO_2_ nanocomposites [34,65,66]. It indicates, that the high electron affinity of NO_2_ gas molecule may result in its enhanced chemisorption on the gold-modified SnO_2_ surface via the same spillover mechanism, which was reported for oxygen molecule [29]. This phenomenon should facilitate complete oxidation of Pd by chemisorbed NO_2_ molecules in case of bimetallic modified AuPd/SnO_2_ nanocomposite, leading to the observed excellent response of this material towards NO_2_.

The performance of Pd/SnO_2_ nanocomposite towards reducing gases in this study is at the same level as for pure SnO_2_ with one exception—detection of CO at low working temperatures. This kind of sensor activity has been described previously for sol-gel derived SnO_2_-Pd nanocomposites and is related to oxygen species transfer between SnO_2_ surface and partially oxidized Pd-clusters at which CO oxidation proceeds via Eley–Rideal mechanism [31]. This mechanism cannot be realized during oxidation of more complex molecules. The fundamental research on the nature of Pd-modification influence on SnO_2_ gas sensing properties revealed the dependence of the effect on the actual Pd content [30,67]. It has been reported, that in case of flame spray derived Pd-loaded SnO_2_ the significant part of Pd is present in Pd^2+^ state at the SnO_2_ lattice cationic positions on the surface and does not participate in sorption processes on the grain’s surface. Thus, the absence of any catalytic effect on Pd/SnO_2_ sensor performance towards reducing gases is observed. The reproducibility of the observed pattern of gas sensor response dependence on sensor working temperature is depicted by Figure S8, where the same measurements with replica sensors are presented. Stability of the metal modified SnO_2_ performance is reflected by Figure S9 with the use of propane as the test gas.

**Figure 6:**
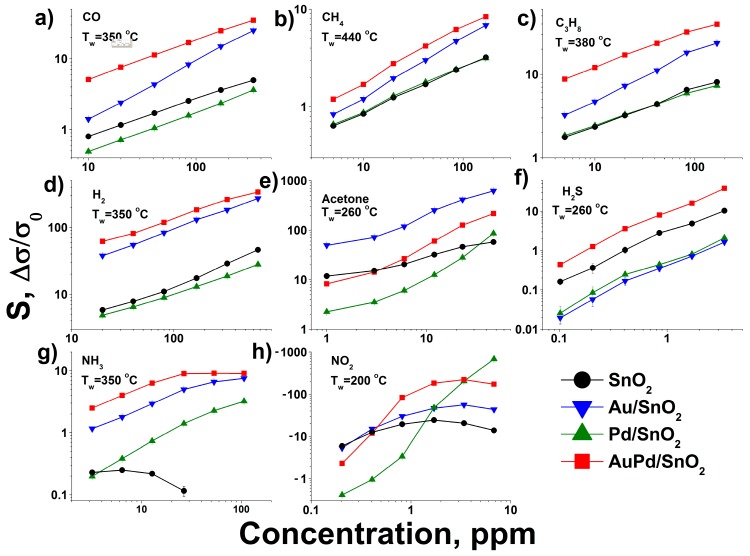
Dependence of gas sensor response of synthesized materials on gas concentration at maximum response working temperature for AuPd/SnO_2_ material: (**a**) CO (**b**) CH_4_ (**c**) C_3_H_8_ (**d**) H_2_ (**e**) acetone (**f**) H_2_S (**g**) NH_3_ (**h**) NO_2_ in dry air.

Concentration dependences of sensor response of synthesized materials towards given gases in dry air at the AuPd/SnO_2_ sample optimum working temperature reveal that this material maintains its excellent sensor performance towards oxidizing and reducing gases in a wide range of concentrations (Figure 6). The few exceptions are acetone and NO_2_. It has already been mentioned that in case of acetone Au/SnO_2_ nanocomposite demonstrates superior sensitivity due to increased amount of active reaction sites on the surface. This effect plays major role in the whole tested range of acetone concentrations. Contrarily, AuPd/SnO_2_ demonstrates lower sensitivity than in case of pure SnO_2_ for acetone below 3 ppm concentration. This may be attributed to the manifestation of electronic Fermi level control effect of metallic Pd on the SnO_2_ resistance, as very poor acetone sensitivity is observed for Pd/SnO_2_ material. The same electronic effect, discussed above, leads to great sensitivity towards high concentrations of NO_2_ for the Pd/SnO_2_ sample. The higher response values of this material, compared to bimetallic AuPd/SnO_2_ sample at NO_2_ concentrations above 5 ppm, should be attributed to higher Pd content. Notably, both Pd-containing materials demonstrate sharp drop in response towards NO_2_ at lower concentrations below 0.5 ppm compared to pure and gold-modified SnO_2_. This picture reflects a complex nature of sensor response of bimetallic AuPd/SnO_2_ material, which have both chemical and electronic components, domination over each other at different analyte concentrations.

The estimated detection limits for the given gases are presented in Table 3. It can be seen that bimetallic modified SnO_2_ demonstrates exceptionally low detection limits in the case of hydrogen and ammonia molecules, while for other gases the detection limits are comparable with other samples. It is required to be underlined that the present values were obtained by extrapolation procedure and may differ from the real values.

**Figure 7:**
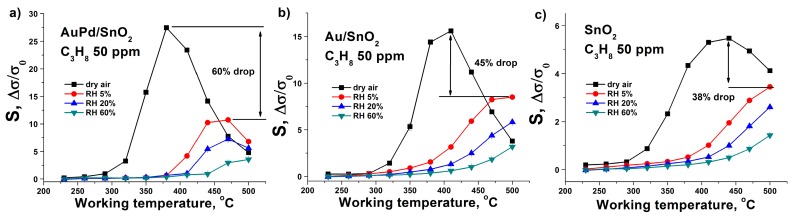
Sensor response dependence on the air humidity for 50 ppm of C_3_H_8_ in the case of (**a**) AuPd/SnO_2_ sample, (**b**) Au/SnO_2_, (**c**) pure SnO_2_ material.

The rise of the air humidity leads to most prominent sensor response drop for AuPd/SnO_2_ material, compared to gold-modified and pure tin dioxide (Figure 7). However, the absolute values of the response for this material are still above the responses of other synthesized samples. Moreover, the highest sensor response for bimetallic modified SnO_2_ is achieved at lower sensor working temperatures in the whole range of tested air relative humidity values.

**Figure 8:**
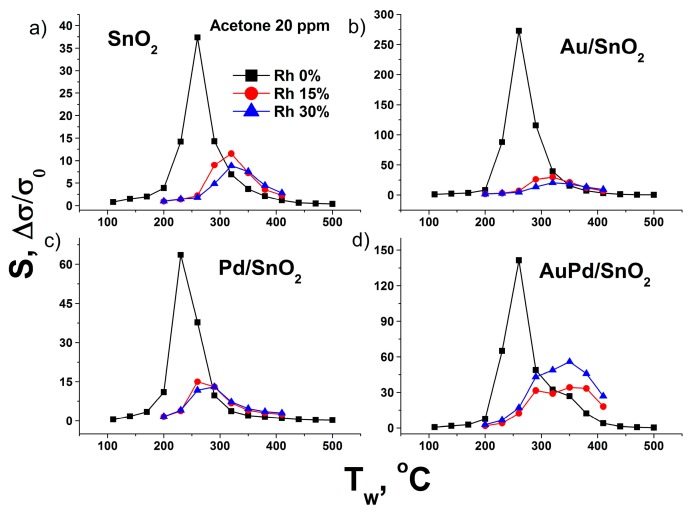
Sensor response dependence on the air humidity for 20 ppm of acetone in the case of (**a**) pure SnO_2_ sample, (**b**) Au/SnO_2_, (**c**) Pd/SnO_2_ and (**d**) AuPd/SnO_2_ material.

The negative effect of humidity is observed in the whole range of working temperatures for all tested gases and leads to gradual decrease of sensor response as air water content grows. The only two observed exceptions are acetone and NO_2_. In the case of acetone (Figure 8) the drop of sensor response after humidity increase from 0 to 15% Rh changes to moderate response growth in case of AuPd/SnO_2_ material after relative humidity increase from 15 to 30%.

This phenomenon may be connected to the participation of dissociated water molecules in the surface oxidation reactions, which is reported for other metal oxides [68], particularly the reoxidation of surface Pd clusters after exposure to reducing gas.

**Figure 9:**
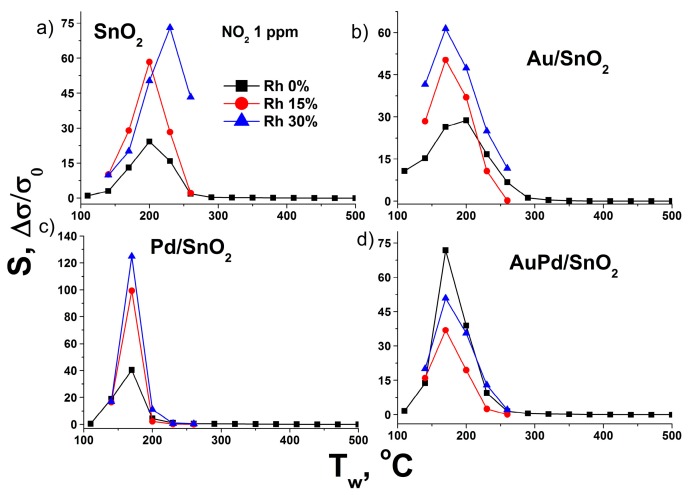
Sensor response dependence on the air humidity for 1 ppm of NO_2_ in the case of (**a**) pure SnO_2_ sample, (**b**) Au/SnO_2_, (**c**) Pd/SnO_2_ and (**d**) AuPd/SnO_2_ material.

Another exception is the humidity effect on the sensor response to NO_2_ which gradually rises with the growth of water content in the air (Figure 9). Such an effect has been reported previously [69] and may be related to favored surface chemisorption of nitrate and nitrite species. Another explanation is the SnO_2_ surface reduction by water molecules [70], which is being reoxidized in the presence of NO_2_ molecules.

Again, the behavior of AuPd/SnO_2_ material differs in this case, demonstrating drop of response on relative humidity increase from 0 to 15%, which changes to moderate growth when relative humidity further increases up to 30%. It indicates the competition between oxygen and NO_2_ molecules for the same active adsorption sites on the surface of Au and Pd clusters, responsible for Pd oxidation to PdO state, which is affected by the presence of water molecules.

**Figure 10:**
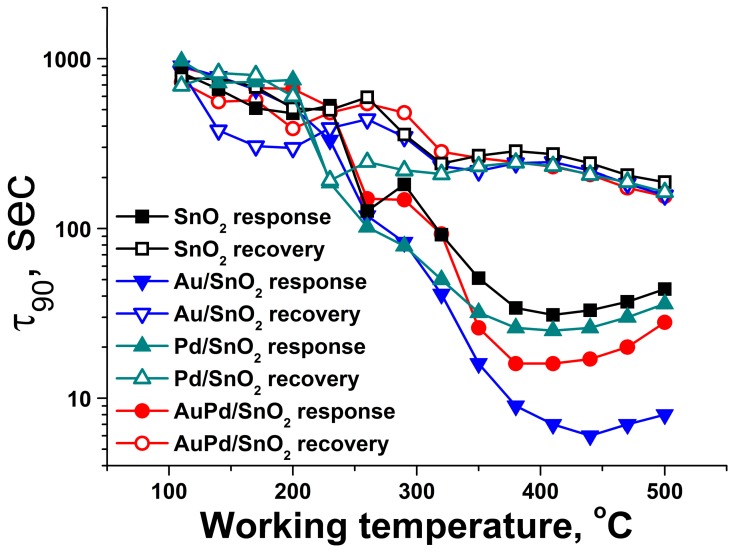
Times of response and recovery for synthesized materials during detection of C3H8 50 ppm in dry air.

Au-containing materials, particularly, the monometallic modified Au/SnO_2_ sample, demonstrate significantly lower times for 90% response during gas sensor operation, compared to pure SnO_2_ (Figure 10). This phenomenon may be related not only to abundance of active sites of gas molecule oxidation on the surface of the material, but also to their higher turnover frequency. However, this matter requires a separate study. The slower response in the case of AuPd/SnO_2_ compared to monometallic Au/SnO_2_ material may be connected to the processes of surface Pd component reduction from PdO to Pd^0^ state, passing at lower rate compared to adsorbed gas molecules oxidation.

## 4. Conclusions

The homogeneous distribution of Au and Pd components over the structure of nanocrystalline SnO_2_ based nanocomposites, achieved by flame spray pyrolysis synthesis technique, gives rise to a superior gas sensor performance of the obtained material. The excellent gas sensor properties arise from synergistic combination of chemical catalytic effect of gold and electronic effect of Fermi level control by surface Pd clusters, prone to switch to PdO state in oxidizing conditions and back to Pd^0^ in the presence of reducing component. Besides being highly effective in achieving of such synergistic effect, FSP is proven to be a convenient technique, which allows to obtain a bimetallic modification of SnO_2_ with Au and Pd components in a single synthetic step with high level of content control.

## Figures and Tables

**Table 1:** Chemical content of noble metals in the composition of the synthesized materials (ICP-MS), average grain size (XRD, Scherrer Equation) and effective surface area (BET).

Sample		Au Load		Pd Load %, Mol	ICP MS, %Mol		d, nm	S_BET,_ m^2^/g
№	Name	%, Mass	% Mol		Au	Pd		
1	SnO_2_	--	--				14	52
2	Au/SnO_2_	0.4	0.31		0.29 ± 0.02		10	70
3	Pd/SnO_2_	--	--	0.31		0.29 ± 0.02	11	69
4	AuPd/SnO_2_	0.2	0.15	0.15	0.16 ± 0.01	0.15 ± 0.01	11	58

**Table 2:** Local atomic content of AuPd/SnO_2_ nanocomposite components (Figure 2).

Spectrum №	Weight Content, %				
	O	Pd	Sn	Au	Total
24	36.59	0	63.41	0	100
25	36.69	2.01	59.59	1.71	100
26	26.81	0.95	71.11	1.14	100
27	34.19	2.77	61.64	1.39	100
28	30.67	1.06	66.82	1.44	100
30	34.71	0	41.38	23.92	100
31	32.96	0	67.04	0	100
32	27.18	0	69.97	2.85	100
33	34.12	3.19	61.76	0.93	100
Map Sum Spectrum	33.23	0.16	66.46	0.15	100

**Table 3:** Limits of detection of the sensors on the basis of the synthesized materials.

Sample	Detection Limit, ppm							
	CO	CH_4_	C_3_H_8_	H_2_	Acetone	H_2_S	NH_3_	NO_2_
SnO_2_	0.002	0.002	0.013	0.007	0.003	0.023	0.41	0.025
Au/SnO_2_	0.04	0.04	0.014	0.0007	0.00013	0.029	0.34	0.03
Pd/SnO_2_	0.002	0.002	0.1	0.004	0.009	0.06	0.38	0.13
AuPd/SnO_2_	0.003	0.003	0.01	0.00006	0.005	0.024	0.025	0.07

## Supplementary Materials

The following are available online at https://www.mdpi.com/2079-4991/9/5/728/s1, Figure S1: Schematic representation of flame spray pyrolysis setup, used for synthesis of nanocomposites, Figure S2: Photographic image of spray nozzle (left) and whole flame spray pyrolysis setup (right) during the process of metal oxide nanocomposites synthesis, Figure S3: XRD pattern of synthesized samples, revealing single phase of tetragonal SnO_2_ in all materials, Figure S4: (a) Particle size distribution histogram for SnO_2_ sample, calculated on the basis of (b) low magnification BF TEM images, Figure S5: (a) Particle size distribution histogram for Pd/SnO_2_ sample, calculated on the basis of (b) low magnification BF TEM images, Figure S6: (a) Particle size distribution histogram for Au/SnO_2_ sample, calculated on the basis of (b) low magnification BF TEM images, Figure S7: (a) Particle size distribution histogram for AuPd/SnO_2_ sample, calculated on the basis of (b) low magnification BF TEM images. Figure S8: Reproducibility of gas sensor response pattern dependence on temperature for replica sensors on the basis of the synthesized materials. Figure S9: Working temperature dependence of gas sensor response of bimetallic modified AuPd/SnO_2_ material towards C_3_H_8_ during 4 weeks of consecutive measurements towards other gases: CO, CH_4_, H_2_, NO_2_, NH_3_, acetone.
