# Supplementary material for: Effect of AuPd Bimetal Sensitization on Gas Sensing Performance of Nanocrystalline SnO2 Obtained by Single Step Flame Spray Pyrolysis
Source: Nanomaterials (Basel). 2019 May 10;9(5):728. doi: 10.3390/nano9050728 (PMC6567076; doi:10.3390/nano9050728)
Supplement: Supplementary file 1 [file nanomaterials-09-00728-s001.pdf]

Supplementary Information

Effect of AuPd Bimetal Sensitization on Gas Sensing Performance of Nanocrystalline SnO_2 Obtained by Single Step Flame Spray Pyrolysis

Valeriy Krivetskiy [1,*], Konstantin Zamanskiy [2], Artemiy Beltyukov [3], Andrey Asachenko [1,4], Maxim Topchiy [1,4], Mikhail Nechaev [1,4], Alexey Garshev [1], Alina Krotova [1], Darya Filatova [1], Konstantin Maslakov [1], Marina Rumyantseva [1] and Alexander Gaskov [1]

[1] Department of Chemistry, Lomonosov Moscow State University, Leninskie gory 1/3, 119234 Moscow, Russia; asandrey@yandex.ru (A.A.); maxtopchiy@ya.ru (M.T.); m.s.nechaev@org.chem.msu.ru (M.N.); garshev@inorg.chem.msu.ru (A.G.); alinakrotova1996@mail.ru (A.K.); gak1.analyt@gmail.com (D.F.); nonvitas@gmail.com (K.M.); roum@inorg.chem.msu.ru (M.R.); gaskov@inorg.chem.msu.ru (A.G.)

[2] Faculty of Materials Sciences, Lomonosov Moscow State University, Leninskie gory 1/3, 119234 Moscow, Russia; zambahrs97@gmail.com

[3] Udmurt Federal Research Center of UB RAS, Laboratory of Atomic Structure and Surface Analysis, Kirova 132, 426000 Izhevsk, Russia; beltukov.a.n@gmail.com

[4] A.V. Topchiev Institute of Petrochemical Synthesis, Russian Academy of Sciences, Leninsky Prospect 29, 119991 Moscow, Russia

* Correspondence: vkrivetsky@inorg.chem.msu.ru

1. Materials Synthesis Setup

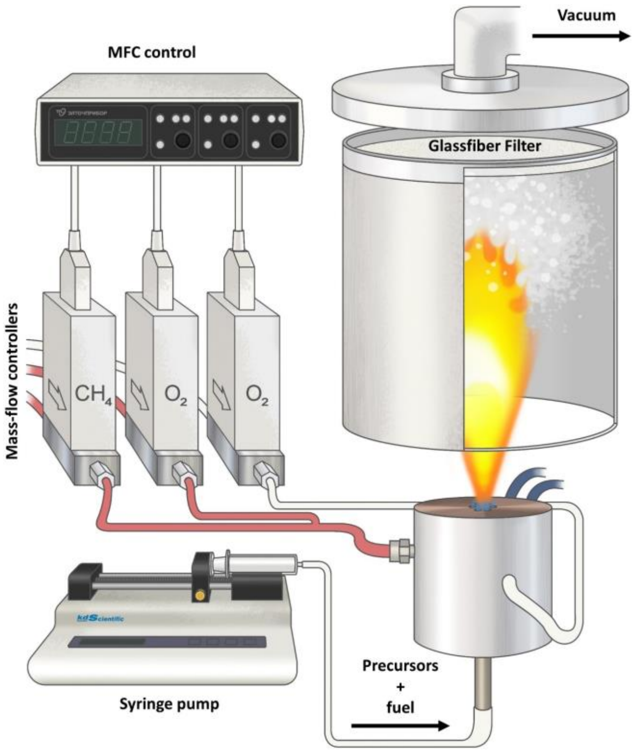

Figure S1. Schematic representation of flame spray pyrolysis setup, used for synthesis of nanocomposites.

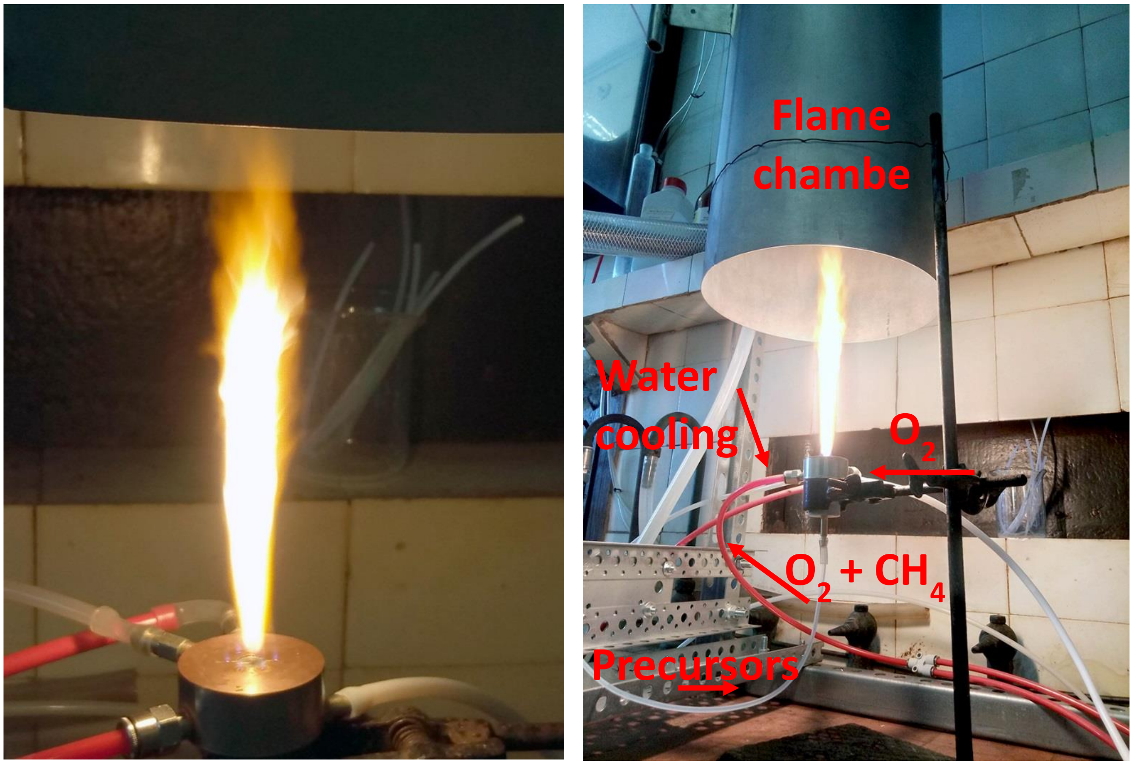

Figure S2. Photographic image of spray nozzle (left) and whole flame spray pyrolysis setup (right) during the process of metal oxide nanocomposites synthesis.

2. Structure and Morphology of the Materials

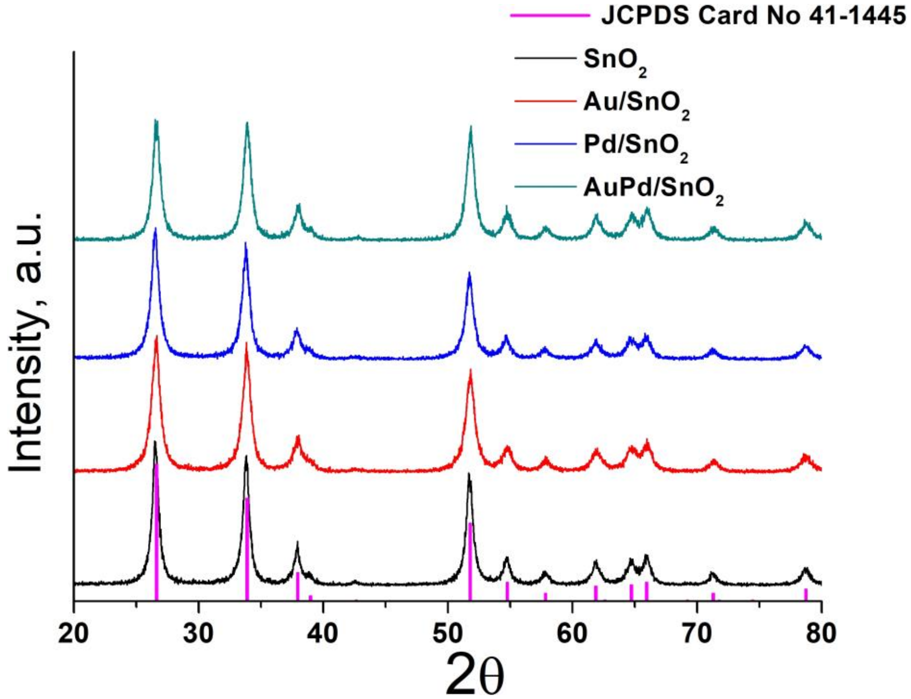

Figure S3. XRD pattern of synthesized samples, revealing single phase of tetragonal SnO_2 in all materials.

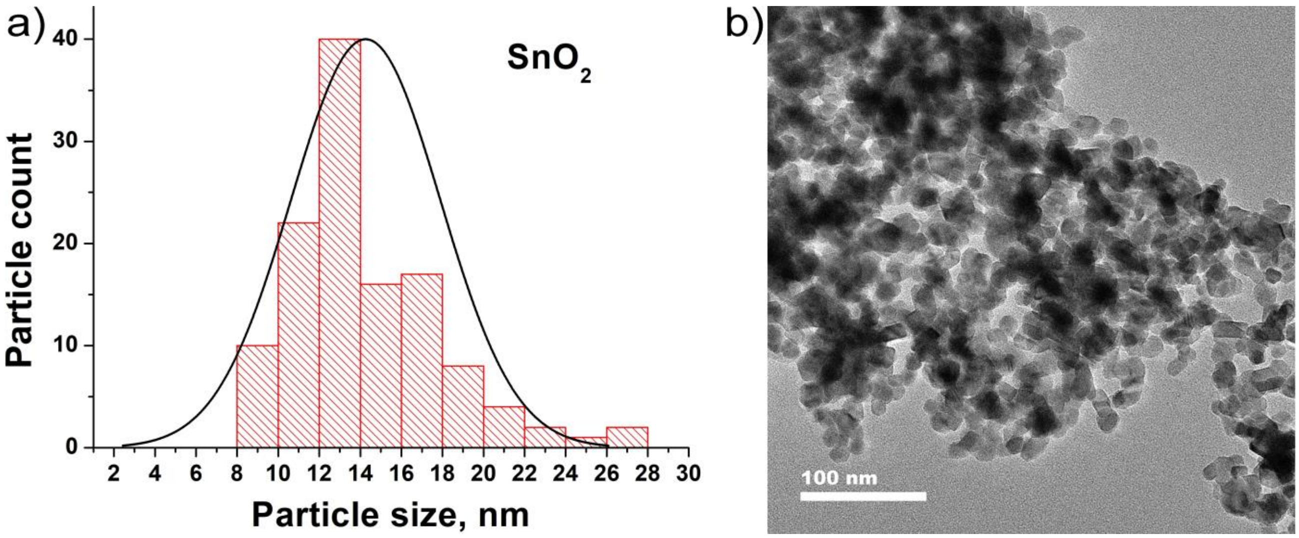

Figure S4. (a) Particle size distribution histogram for SnO_2 sample, calculated on the basis of (b) low magnification BF TEM images.

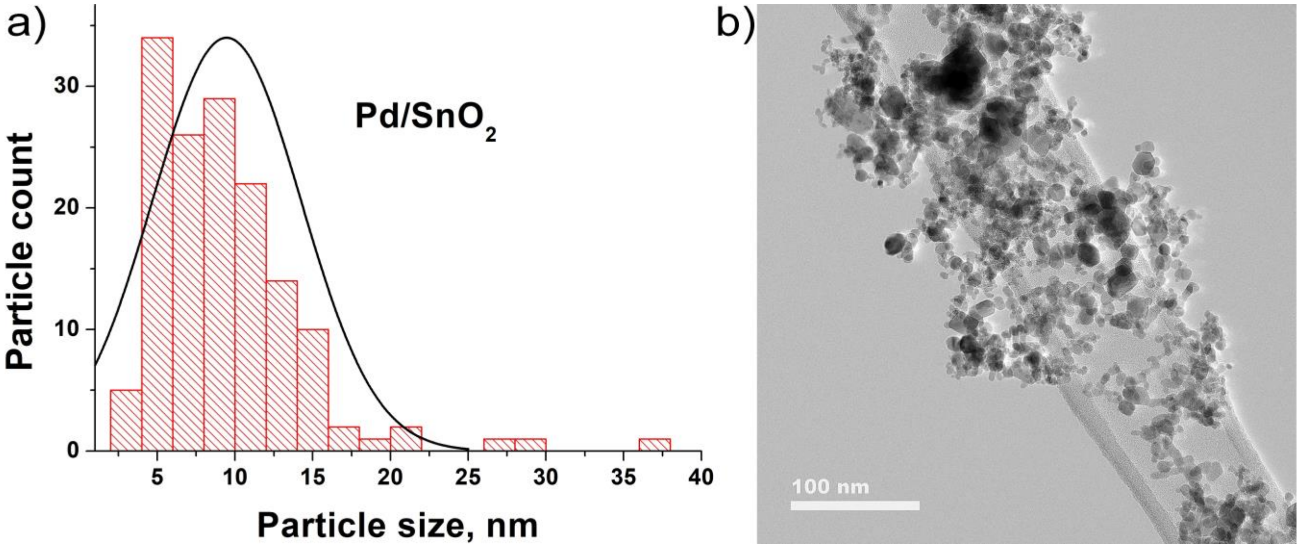

Figure S5. (**a**) Particle size distribution histogram for Pd/SnO_2 sample, calculated on the basis of (**b**) low magnification BF TEM images.

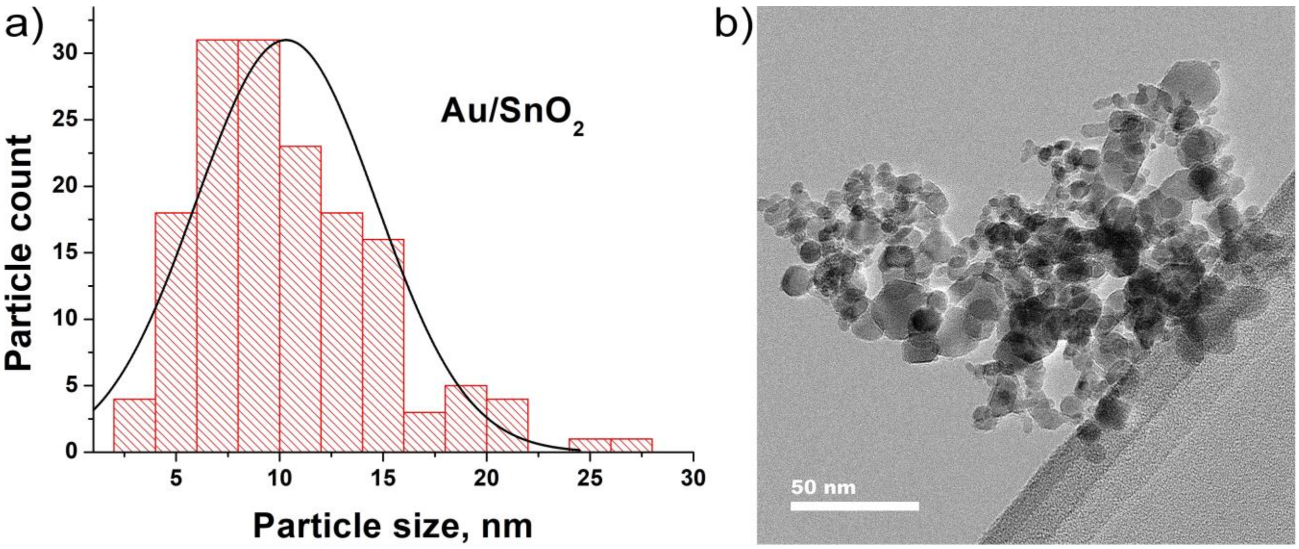

Figure S6. (**a**) Particle size distribution histogram for Au/SnO_2 sample, calculated on the basis of (**b**) low magnification BF TEM images.

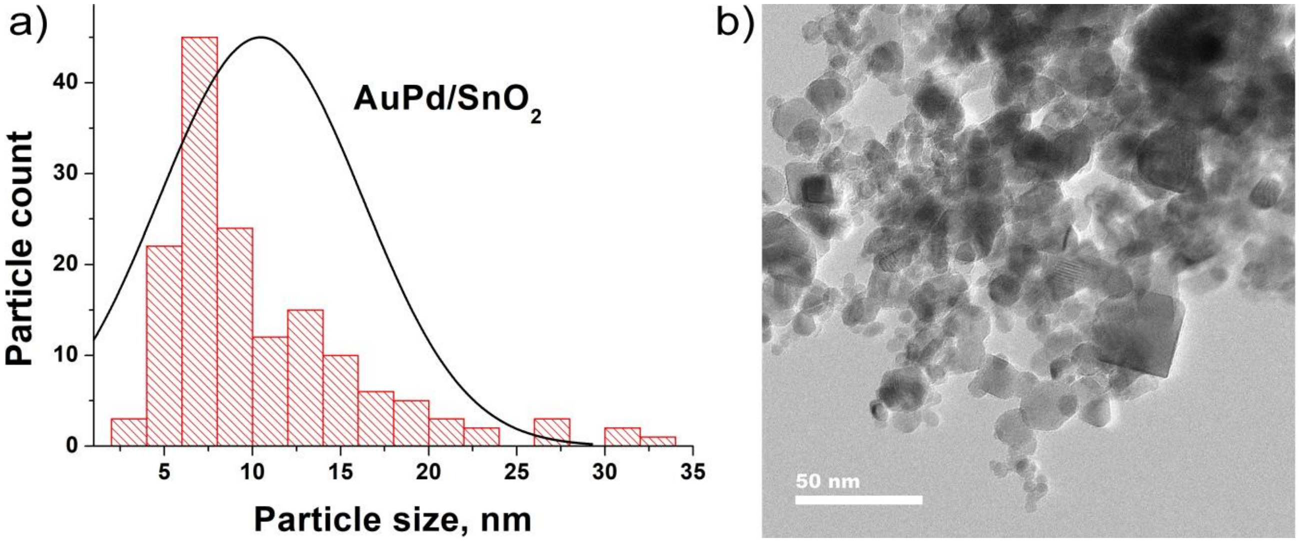

Figure S7. (**a**) Particle size distribution histogram for AuPd/SnO_2 sample, calculated on the basis of (**b**) low magnification BF TEM images.

3. Gas Sensor Properties

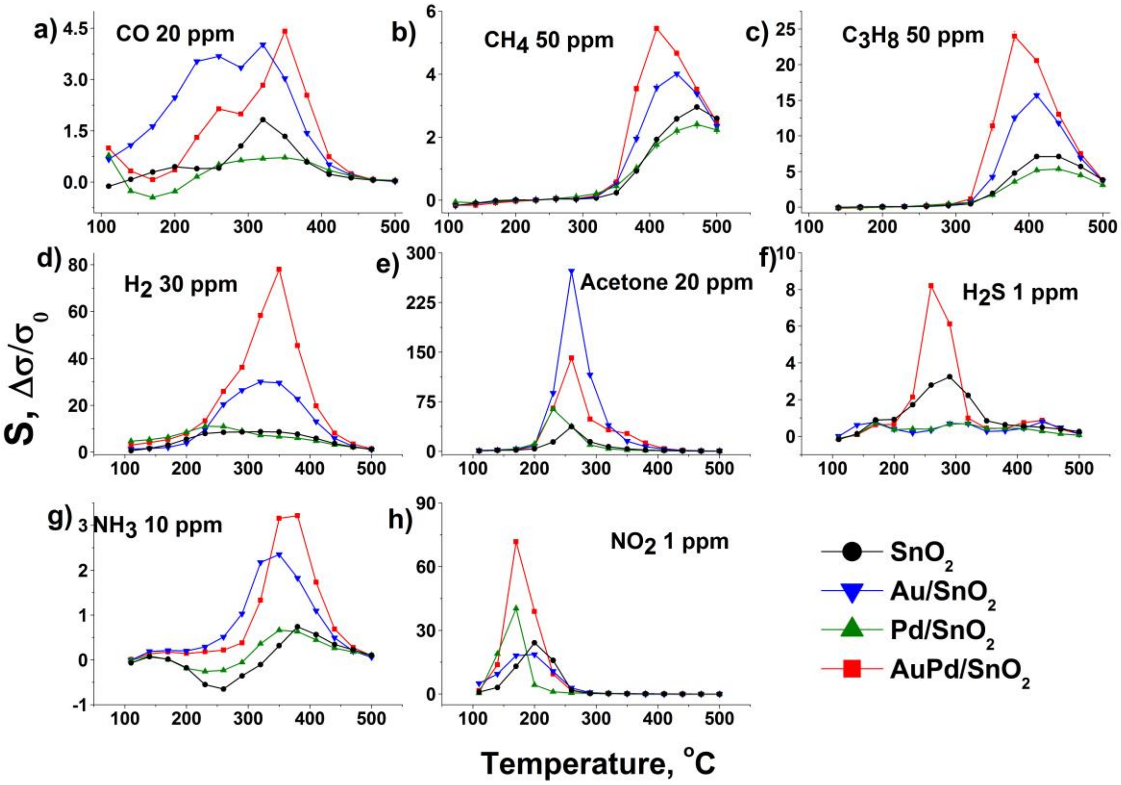

Figure S8. Reproducibility of gas sensor response pattern dependence on temperature for replica sensors on the basis of the synthesized materials.

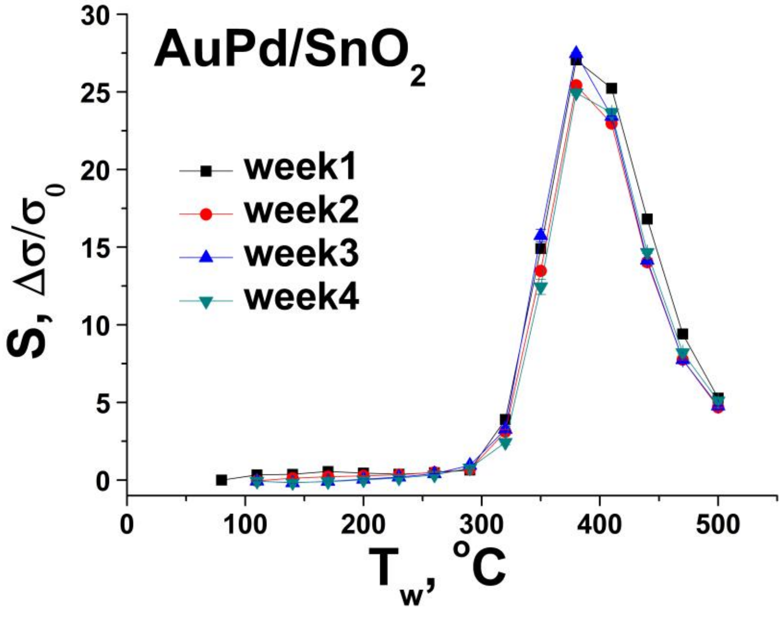

Figure S9. Working temperature dependence of gas sensor response of bimetallic modified AuPd/SnO_2 material towards C_3H_8 during 4 weeks of consecutive measurements towards other gases: CO, CH_4, H_2, NO_2, NH_3, acetone.
